# Solar-powered multi-organism symbiont mimic system for beyond natural synthesis of polypeptides from CO_2_ and N_2_

**DOI:** 10.1126/sciadv.adf6772

**Published:** 2023-03-15

**Authors:** Wen Yu, Yue Zeng, Zenghao Wang, Shengpeng Xia, Zhiwen Yang, Weijian Chen, Yiming Huang, Fengting Lv, Haotian Bai, Shu Wang

**Affiliations:** ^1^ Beijing National Laboratory for Molecular Sciences, Key Laboratory of Organic Solids, Institute of Chemistry, Chinese Academy of Sciences, Beijing 100190, P. R. China.; ^2^ College of Chemistry, University of Chinese Academy of Sciences, Beijing 100049, P. R. China.

## Abstract

Developing artificial symbionts beyond natural synthesis limitations would bring revolutionary contributions to agriculture, medicine, environment, etc. Here, we initiated a solar-driven multi-organism symbiont, which was assembled by the CO_2_ fixation module of *Synechocystis* sp., N_2_ fixation module of *Rhodopseudomonas palustris*, biofunctional polypeptides synthesis module of *Bacillus licheniformis*, and the electron transfer module of conductive cationic poly(fluorene-*co*-phenylene) derivative. The modular design broke the pathway to synthesize γ-polyglutamic acid (γ-PGA) using CO_2_ and N_2_, attributing to the artificially constructed direct interspecific substance and electron transfer. So, the intracellular ATP and NADPH were enhanced by 69 and 30%, respectively, and the produced γ-PGA was enhanced by 104%. The strategy was further extended to produce a commercial antibiotic of bacitracin A. These achievements improve the selectivity and yield of functional polypeptides with one click by CO_2_ and N_2_, and also provide an innovative strategy for creating photosynthetic systems on demand.

## Teaser

A solar-powered multi-organism symbiont system based on energy transfer is constructed to synthesize polypeptides from CO_2_ and N_2_.

## INTRODUCTION

Carbon neutrality is broadly recognized as a vehicle for climate action and sustainable development due to the markedly increased carbon dioxide (CO_2_) concentration caused by the utilization of fossil fuels and human activities *(**1*–*3*). While some outstanding progress referring to the catalytic conversion of CO_2_ into simple C1 and C2 products driven by renewable electricity or solar energy has been completed (*4*, *5*), sustainable conversion of CO_2_ into high value-added longer-chain products has important technical and social implications (*6*–*9*). Recently, a hybrid chemobiological pathway has been demonstrated to synthesize starch from CO_2_ and H_2_ through 11 core reactions relying on expensive enzyme catalysts and stringent carbon conversion conditions (*10*). Zheng *et al.* (*11*) converted CO_2_ into glucose and fatty acid through electrochemical and metabolic engineering. Moreover, nitrogen immobilization plays a crucial role in the biogeochemical cycle, with notable impacts on the agricultural and food industries (*12*). The symbiotic nitrogen fixation with microorganisms in plants is the largest natural bioavailable nitrogen source in the biosphere (*13*). Biological symbiosis system is also an effective way for carbon sequestration by supporting complementary metabolism and conversion of CO_2_ through intimate electronic and substance communications (*14*, *15*). Unfortunately, the low efficiency of electronic and substance communications between species results in an unsatisfying low atom economy (*16*–*20*). Thus, developing artificial symbionts that mimic how symbiotic algae and microorganisms fix CO_2_ and nitrogen to produce proteins or polypeptides and go beyond natural synthesis limitations may bring revolutionary contributions to the fields of agriculture, medicine, environment, and food.

**Fig. 1.**
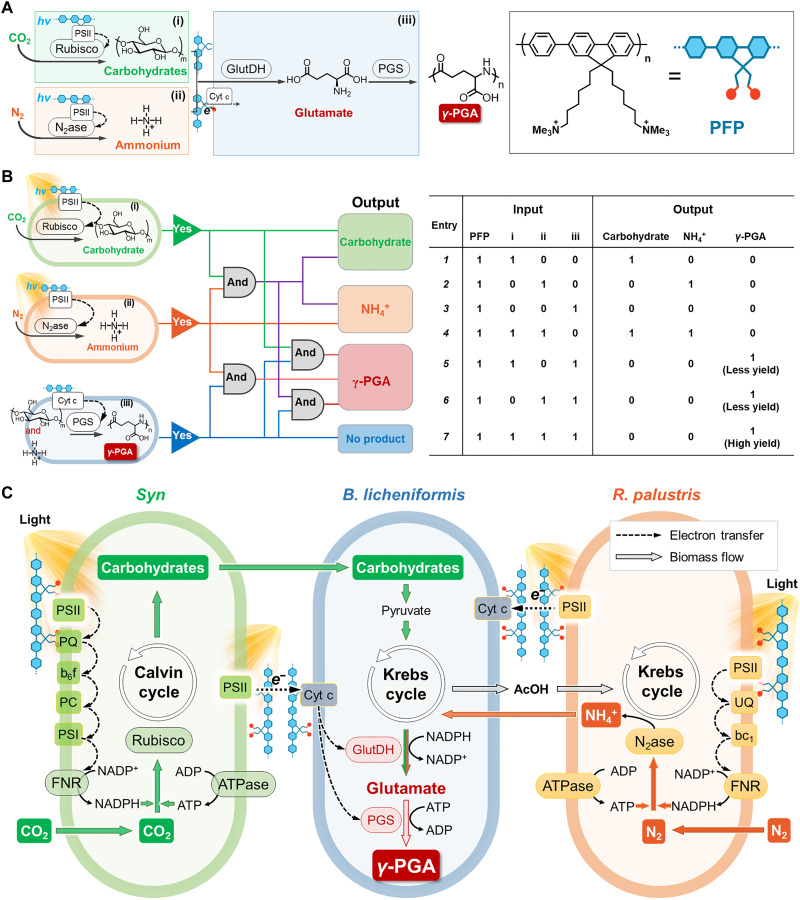
Solar-powered multi-organism symbiont mimic system for beyond natural synthesis of polypeptides. (**A**) Design and modular assembly of γ-PGA biosynthesis pathway. (**B**) Logical biosynthetic circuit from CO_2_ and N_2_. (i) Reduction of CO_2_ to carbohydrates by *Syn*. (ii) Fixation of N_2_ to ammonium by *R. palustris*. (iii) γ-PGA synthesis from carbohydrates and ammonium by *B. licheniformis*. The main production of logical biosynthesis for different modules combined. (**C**) Schematic diagram of electron and substance transfer path in coculture system. PSII, photosystem II; PSI, photosystem I; PQ, plastoquinone; b_6_f, cytochrome b_6_f; PC, plastocyanin; FNR, ferredoxin-NADP^+^ reductase; bc_1_, cytochrome bc_1_; UQ, ubiquinone; Rubisco, ribulose bisphosphate carboxylase oxygenase; Cyt c, cytochrome c; N_2_ase, nitrogenase; GlutDH, glutamate dehydrogenase; PGS, polyglutamate synthetase.

In this work, we initiate a solar-driven multi-organism symbiont for the selective synthesis of functional polypeptides using airborne CO_2_ and N_2_ as carbon and nitrogen sources based on enhanced direct interspecific substance and electron transfer (DISET) through conducting polymers (Fig. 1A). In the system, the modularity enables the variation and synergy of input and logical functions used to produce a theoretically infinite variety of biochemicals (Fig. 1B and fig. S1). The γ-polyglutamic acid (γ-PGA) and bacitracin A are selected as target polypeptides due to their low biosynthesis efficiencies. γ-PGA is a polypeptide widely used in the food industry, pharmacy, environmental protection, and agriculture, while bacitracin A is a kind of polypeptide antibiotic (*21*–*23*). The conception of the solar-driven symbiont is adopted to couple the CO_2_ fixation module of *Synechocystis* sp. *PCC6803* (*Syn*), N_2_ fixation module of *Rhodopseudomonas palustris*, γ-PGA synthesis module of *Bacillus licheniformis*, and the electron transfer module of conductive polymer [PFP: cationic poly(fluorene-*co*-phenylene) derivative] (*24*, *25*). Conductive polymers are filled with the advantages of adjustable bandgap, good biocompatibility, and good conductivity (*26*–*30*). They had been reported to improve photosynthesis (*31*, *32*), promote biomass conversion (*33**)*, and improve photoelectric efficiency by hybridization with biological systems (*34*). Thus, positively charged PFP could form a conductive network by making the three organisms gather and promote direct interspecific electron transfer due to its excellent conductivity. Furthermore, PFP with excellent light capture ability could augment the photosynthesis of *Syn* and *R. palustris*, thus enhancing the fixation of CO_2_ and N_2_. *B. licheniformis* can further transform the generated carbohydrate and NH_4_^+^ into γ-PGA biopolymer through the bacterial metabolic pathway.

## RESULTS

The controlled synthesis of γ-PGA using CO_2_ and N_2_ through detailed electron, substance transfer, and synthesis pathways is shown in Fig. 1C. For the carbon fixation module, *Syn* converts CO_2_ into carbohydrates through ribulose-1,5-diphosphate carboxylase/oxygenase (Rubisco), and for the nitrogen fixation module, *R. palustris* fixes N_2_ through nitrilase (N_2_ase) into dissolving NH_4_^+^. For the electron transfer module, conductive polymer PFP shortens the distance among species by aggregating them to form a conduction network. By expanding the light capture into the ultraviolet (UV) range by PFP, the photosynthetic efficiency and photogenerated electron’s ability of *Syn* and *R. palustris* are both enhanced. The photogenerated electrons are transmitted to *B. licheniformis* for synthesis module through PFP to improve the yields of intracellular adenosine triphosphate (ATP) and NADPH [reduced form of nicotinamide adenine dinucleotide phosphate (NADP^+^)]. *B. licheniformis* uses carbohydrates and NH_4_^+^ to synthesize γ-PGA through the metabolic pathway in a controllable and programmable manner. This method is easily extended to the synthesis of antibiotic bacitracin A using CO_2_ and N_2_ as carbon and nitrogen sources through DISET among organism species with different input instructions.

### The interaction between PFP and organisms

Confocal laser scanning microscopy (CLSM) and scanning electron microscopy (SEM) were performed to directly observe the interactions between the three organisms and PFP, respectively. After coincubation with PFP, the blue signals performed on the three organisms are from the fluorescent emission of PFP, and their surfaces were also much rougher than the PFP-free groups in SEM images (figs. S2 and S3). Zeta potential and isothermal microthermal titration (ITC) were measured to explore their binding modes. The zeta potentials of *Syn*, *R. palustris*, and *B. licheniformis* were −51, −37, and −44 mV, respectively, while their zeta potentials were all positively shifted to −23, −28, and −32 mV, respectively, after incubation with PFP (fig. S4A). The ITC results showed that the binding constants (*K*_a_) of *Syn*/PFP and *R. palustris*/PFP were 2.59 × 10^5^ and 2.15 × 10^5^ M^−1^ (fig. S4, B and D), respectively. Hydrophobic fusion existed between positive entropy and positive enthalpy change (*35*, *36*). The values of TΔ*S* were greater than Δ*H*, indicating that the interaction between PFP, *Syn*, and *R. palustris* was entropy driven. The increase of entropy was mainly due to the increase of disordered water molecules in the system. Therefore, the interaction between the hydrophobic side chain of PFP and the outer membrane of *Syn* and *R. palustris* is mainly hydrophobic fusion. The initial negative enthalpy of *B. licheniformis*/PFP indicated the electrostatic interactions. After reaching saturation, the enthalpy change gradually decreased and finally approached zero when PFP was added continuously (fig. S4C), which demonstrated the hydrophobic action in the second half of the process. The *K*_a_ values of *B. licheniformis*/PFP in two stages were calculated as 1.56 × 10^8^ and 6.61 × 10^6^ M^−1^, respectively. The early stage of the interaction was the electrostatic interaction between positively charged PFP and negatively charged outer membrane of *B. licheniformis*. Then, the hydrophobic side chain of PFP was combined with the outer membrane of *B. licheniformis* through hydrophobic interaction. These results showed that PFP could stably bind upon the membranes of all three kinds/organisms by electrostatic and hydrophobic interactions.

### The promotion of the activity of organisms by PFP

**Fig. 2.**
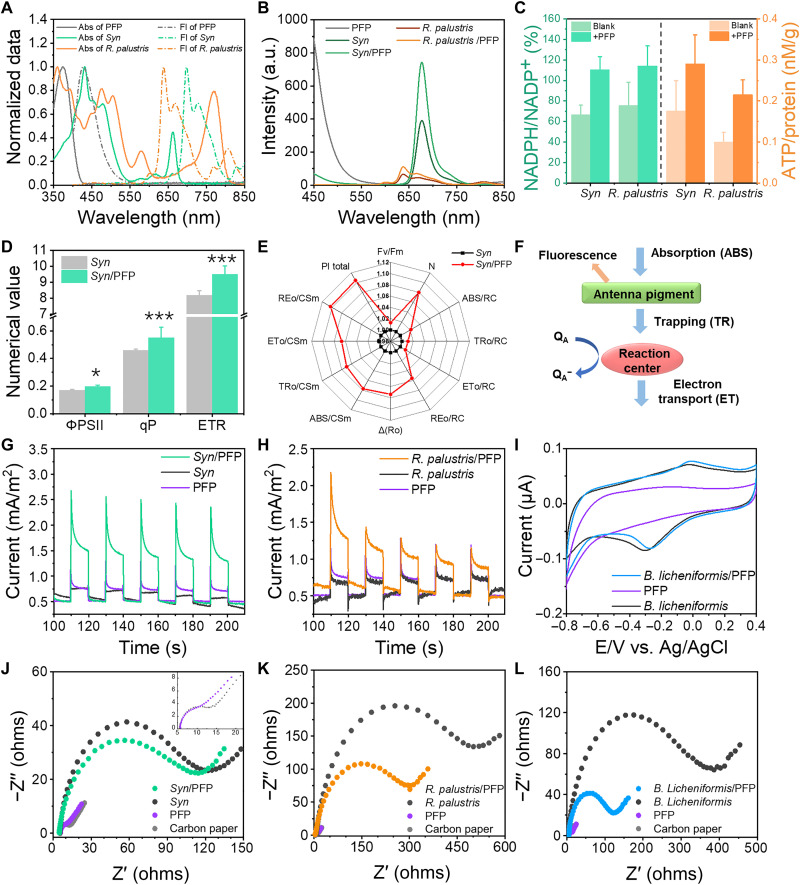
The promotion of the activity of *Syn*, *R. palustris*, and *B. licheniformis* by PFP. (**A**) Normalized UV-visible absorption and fluorescence spectra of PFP, *Syn*, and *R. palustris* (λ_ex_ = 380, 440, and 470 nm, respectively). (**B**) Fluorescence excitation spectra of PFP, *Syn*, *Syn*/PFP, *R. palustris*, and *R. palustris*/PFP with the excitation of 400 nm. (**C**) NADPH/NADP^+^ and ATP contents of *Syn*, *Syn*/PFP, *R. palustris*, and *R. palustris*/PFP. (**D**) Chlorophyll fluorescence parameters of *Syn* and *Syn*/PFP under light adaption. (**E**) Spider plots of chlorophyll fluorescence parameters of *Syn* and *Syn*/PFP under dark adaptation. (**F**) Simplified scheme for energy cascade from light absorption to electron transport. (**G**) Photocurrent responses of *Syn*, PFP, and *Syn*/PFP. (**H**) Photocurrent responses of *R. palustris*, PFP, and *R. palustris*/PFP. (**I**) Cyclic voltammetries of *B. licheniformis*, PFP, and *B. licheniformis*/PFP in anaerobic environment. (**J**) Electrochemical impedance spectroscopy (EIS) measurements of *Syn* and *Syn*/PFP electrodes. (**K**) EIS measurements of *B. licheniformis* and *B. licheniformis*/PFP electrodes. (**L**) EIS measurements of *R. palustris* and *R. palustris*/PFP electrodes. a.u., arbitrary units.

The absorption spectra (330 to 430 nm) of PFP were complementary to those of the photosynthetic *Syn* and *R. palustris*, while PFP emission (400 to 550 nm) overlapped with absorption spectra of *Syn* and *R. palustris* (Fig. 2A). In the *Syn*/PFP and *R. palustris*/PFP pairs, the fluorescence intensity of PFP around 450 nm was decreased, while the fluorescence of *Syn* and *R. palustris* was notably enhanced around 680 and 640 nm (Fig. 2B). It demonstrated that the irradiation energy in the range of 330 to 430 nm could be captured by PFP and transferred to *Syn* or *R. palustris*. Then, *Syn* and *R. palustris* assembled with PFP respectively performed increased NADPH/NADP^+^ ratios and ATP content by 52 and 66% before and after PFP introduction (Fig. 2C). The fluorescence parameters of chlorophyll were used to determine the photochemical and nonphotochemical processes of *Syn* antenna molecules after light or dark adaptation. As shown in Fig. 2D, the photochemical efficiency of *Syn* (ϕPSII), photochemical quenching coefficient (qP), and electron transfer rate were increased by 15.9, 19.0, and 15.9%, respectively, after binding with PFP. In addition, the increased ratios of F_v_/F_m_ (maximal quantum yield of PSII under dark adaptation), Pl total (comprehensive performance index), ABS/RC (absorbed energy per reaction center), TR_o_/RC (trapped energy per reaction center), ET_o_/RC (energy used for electron transfer per reaction center), RE_o_/RC (energy transported to PSI per reaction center), ABS/CS_m_ (absorbed energy per unit area), TR_o_/CS_m_ (trapped energy per unit area), ET_o_/CS_m_ (energy used for electron per unit area), RE_o_/CS_m_ (energy transported per unit area), and Δ(R_o_) (the efficiency of electron transfer to PSI). showed that the energy absorption, capture, and electron transfer chain efficiency of *Syn*/PFP were all enhanced than the free *Syn* (Fig. 2E). Referring to the standard electron transfer and energy cascade of photosynthetic process (Fig. 2F), all the above results demonstrated that PFP could enhance photosynthetic efficiency of PSII (light absorption, capture, utilization, and electron transfer). The improved light energy utilization and photosynthetic products of *Syn* and *R. palustris* could supplymore substances for the next synthesis step.

Because *B. licheniformis* could use the external electrons to produce γ-PGA, the improved generation and transfer of photoelectrons were subsequently studied by chronoamperometry. As shown in Fig. 2 (G and H), *Syn*/PFP electrode (0.92 mA/m^2^) and *R. palustris*/PFP electrode (0.53 mA/m^2^) exhibited more substantial photocurrent enhancement than the *syn* electrode (0.19 mA/m^2^) and *R. palustris* electrode (0.20 mA/m^2^) under white light illumination. As per the cyclic voltammetry of *B. licheniformis* and *B. licheniformis*/PFP shown in Fig. 2I, the obvious redox peaks at −0.25 and 0.05 V indicated that the direct electron transport redox proteins (Cyt c) on the surface of *B. licheniformis* was not disturbed by PFP, and Cyt c could also be worked as the medium for electron transfer from *Syn* and *R. palustris* to *B. licheniformis.* Moreover, *R*_ct_ of organism electrodes of *Syn*, *B. licheniformis*, and *R. palustris* binding with PFP were respectively decreased from 125, 390, and 550 ohms to 110, 125, and 205 ohms (Fig. 2, J to L). The growth curves additionally confirmed the good biocompatibility of PFP in the microbial system (fig. S5, A to C). These results characterized that PFP could further improve the conductivity of all the three organisms and accelerate the electron transfer between them, except for the increased photosynthetic products.

### The construction of multi-organism symbiont mimic system

The morphology analysis characterized the formation of the multi-organism assemblies consisting of the three organisms and PFP. As per the CLSM, SEM, and atomic force microscopy (AFM) images shown in figs. S6 to S8, the observed blue fluorescent signal, coarser bacterial surfaces, and certain height of bulge on bacterial cells indicated that PFP was evenly distributed in the organism clusters (figs. S6 to S8). The closed connection between organisms would favor substance communication, and the effect of direct interspecific electron transfer was investigated by Kelvin probe force microscopy. As shown in fig. S9, the potential difference between the bacterial cluster with PFP and the substrate was 5 mV, which was much smaller than the control group without PFP (22 mV). It means that PFP with satisfying conductivity could contribute to the better direct interspecific electron transfer by forming a network of assembled organisms.

**Fig. 3.**
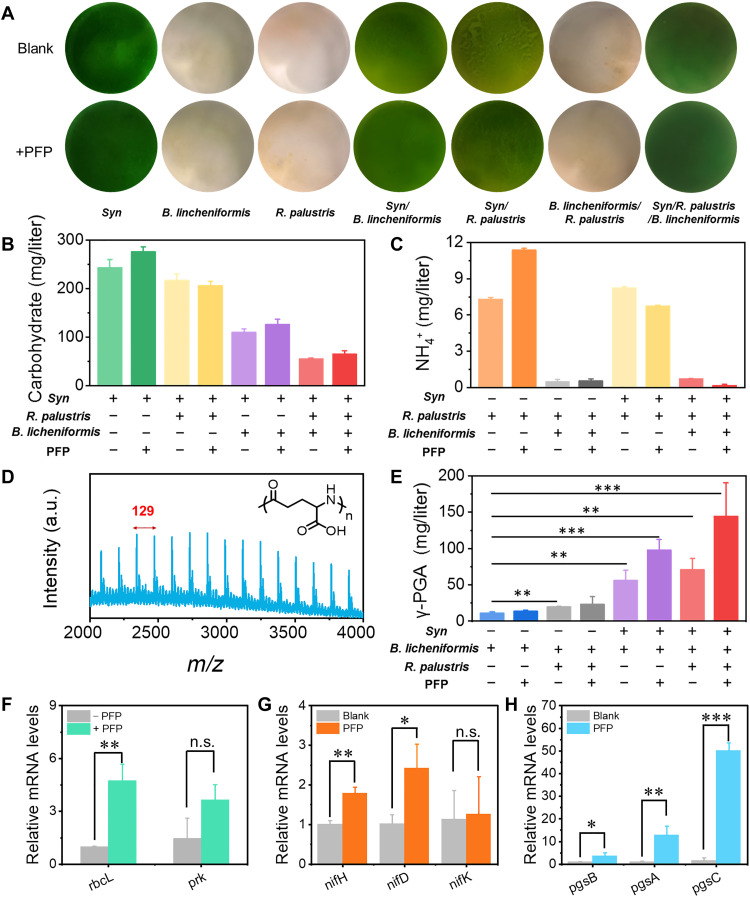
The construction and characterization of multi-organism symbiont mimic system for synthesis of γ-PGA. (**A**) Appearance of coculture system with different components after growing for 10 days. (**B**) Carbohydrate production of different logic synthesis circuits after growing for 10 days. (**C**) NH_4_^+^ production of different logic synthesis circuits after growing for 10 days. (**D**) Mass spectrometry of γ-PGA produced by *Syn*/*R. palustris*/*B. licheniformis*/PFP. (**E**) Synthetic γ-PGA production of different logic synthesis circuits after growing for 10 days. (**F** to **H**) Relative mRNA expression level of *rbcL*, *prk*, *nifH*, *nifD*, *nifK*, *pgsB*, *pgsA*, and *pgsC *in *Syn*/*R. palustris*/*B. licheniformis* cultured in PFP. *P < 0.05, **P < 0.01, and ***P < 0.001 relative to control. n.s., not significant.

Subsequently, the most important symbiotic relationship and certain influence of *Syn*, *B. licheniformis*, and *R. palustris* were investigated by observing the growth situation. As shown in Fig. 3A, all the multi-organism systems could coexist and grow well together as long as the photoautotrophic *Syn* was contained, while *B. licheniformis* and *R. palustris* could not survive on their own. Moreover, PFP have no adverse impact on the symbiont. According to the carbohydrate concentration in Fig. 3B, all the cocultured systems containing *Syn* could fix and convert CO_2_ due to the photosynthetic process. The calculation results showed that the carbohydrate of *Syn* alone was 242.9 mg/liter, and it was further increased by 14% to 275.7 mg/liter after adding PFP. For both *B. licheniformis* and *R. palustris* to reduce the carbohydrate generated by *Syn*, the carbohydrate concentration was decreased to 65 and 55.4 mg/liter in three-organism symbiont with or without PFP. It means that the consumed carbohydrate in three-organism systems was increased from 186.6 to 210.6 mg/liter after adding PFP. The other two photosynthetic carbon fixation units of *Chlorella pyrenoidosa* and *Synechococcus* were also respectively cocultured with *B. licheniformis* instead of *Syn*. However, they could not coexist with *B. licheniformis* because of the quorum sensing and interactions. The selected *Syn* was expected to form the symbiotic relationship for γ-PGA synthesis. In addition to the carbohydrate, the abundant ammoniums from *R. palustris* were another direct element for confirming the γ-PGA generation. Similarly, the NH_4_^+^ yield produced by photosynthetic *R. palustris* was increased from 7.3 to 11.4 mg/liter with the addition of PFP (Fig. 3C). In addition, the NH_4_^+^ content decreased substantially in all the multi-organism groups because both *Syn* and *B. licheniformis* could absorb the generated NH_4_^+^ for their growth and metabolism. Especially for the cocultured systems containing *B. licheniformis*, the concentration of NH_4_^+^ was reduced to less than 0.70 mg/liter because of the γ-PGA biosynthesis process inside. The further quantitative analysis showed that the NH_4_^+^ consumed in *Syn*/*R. palustris*/*B. licheniformis* was increased from 6.61 to 11.22 mg/liter in the presence of PFP. Moreover, *R. palustris* could oxidize the acetic acid to generate electrons except for the NH_4_^+^, while the acetic acid was coincidently one of the by-products of *B. licheniformis* during γ-PGA production (fig. S10). Therefore, the acetic acid concentration in the *Syn*/*B. licheniformis* system decreased from 5.43 to 1.83 mM after the introduction of *R. palustris*. To sum up, *Syn*, *R. palustris*, and *B. licheniformis*, with mutually beneficial relationships, formed an artificial symbiotic system by PFP, where PFP could promote the carbon and nitrogen source generation, communication, and utilization for γ-PGA synthesis efficiently.

The increased electron transfer between bacterial species by PFP was another reasonable factor for the more γ-PGA generation. So two-chamber microbial fuel cells (MFCs) were fabricated to determine the influence of PFP on direct interspecific electron transfer efficiency (fig. S11). The maximum current density and power of *Syn*/*B. licheniformis* were respectively increased from 11.8 μA/cm^2^ and 1.82 μW/cm^2^ to 20.8 μA/cm^2^ and 4.78 μW/cm^2^ once PFP was added. The MFC of *R. palustris* and *B. licheniformis* with PFP performed a similar increased tendency, which was increased from 11.2 μA/cm^2^ and 0.52 μW/cm^2^ to 14.7 μA/cm^2^ and 1.95 μW/cm^2^. Meanwhile, all the power of MFCs was substantially decreased when the organisms were dead, and the electron transfer between *Syn* and *R. palustris* was hardly observed (fig. S11, E and F). The MFC of *R. palustris* and *B. licheniformis* increased from 2.30 to 4.65 μW/cm^2^ with the acetic acid due to the good ability of *R. palustris*/PFP to oxidize acetic acid (fig. S12). The increase of biocurrents depended on the enhancement of photogenerated electrons and the improvement of electron transport ability. PFP enhanced *Syn* and *R. palustris* photosynthesis to produce more photoelectrons. Meanwhile, conductive polymer could also form the conductive network, and it is beneficial for the efficient electron transfer between microorganisms and electrodes (*37*). Therefore, PFP could improve the photoelectron generation from *Syn* and *R. palustris* and accelerate the direct electron transfer in the multi-organism symbiont. As a result, the NADPH/NADP^+^ and ATP levels of *Syn*/*B. licheniformis*/*R. palustris*/PFP system were increased around 30 and 69%, respectively, compared with the control groups without PFP (fig. S13).

The successful generation of γ-PGA in the constructed symbiont was verified by the standard glutamate monomer spacing of 129 in the mass spectrometry, and the advantaged symbiotic effect was further detailed by quantitative analysis in Fig. 3 (D and E) and fig. S14. The γ-PGA concentration of *B. licheniformis* was only about 10.7 mg/liter, while those in the symbiotic system of *Syn*/*B. licheniformis* and *Syn*/*R. palustris*/*B. licheniformis* were increased to 55.9 and 70.7 mg/liter. After assembly with PFP, the γ-PGA yield in *Syn*/*B. licheniformis*/PFP pair was further increased to 97.8 mg/liter and finally arrived to 144.2 mg/liter (104% increase) in *Syn*/*R. palustris*/*B. licheniformis*/PFP, although the *B. licheniformis*/PFP group had a similar concentration of γ-PGA as the *B. licheniformis* alone.

Photosynthetic efficiency is quantified by the ratio of the available electrons used for production to the total input photon flux (table S2) (*38*). After the addition of PFP, the photosynthetic efficiency of N_2_ conversion to NH_4_^+^ increased from 0.20 to 0.31% in our system, and that of the CO_2_ conversion to carbohydrate increased from 1.97 to 2.23%. The introduction of PFP increased the conversion rate of γ-PGA from 36 to 64%. The photosynthetic efficiency of artificial solar-powered symbiont system with the introduction of PFP increased from 0.71 to 1.43%, which further indicated that PFP could promote the photosynthetic efficiency and product selectivity of symbiont by expanding the light trapping range and accelerating electron transfer. To further expose the mechanism in the symbiont of multi-organisms and PFP, the expression levels of key genes related to CO_2_ fixation, N_2_ conversation, and γ-PGA synthesis were analyzed by real-time quantitative reverse transcription polymerase chain reaction (RT-PCR). The expression levels of *rbcL* and *prk*, which are the subunits of Rubisco and essential enzyme in the Calvin cycle, are up-regulated by 374 and 148%, respectively, in *Syn* after adding PFP (Fig. 3F). Similarly, *nifH* and *nifD* of N_2_ase were up-regulated by 79 and 141% in *R. palustris*/PFP compared with the free *R. palustris* (Fig. 3G). The *pgsB*, *pgsA*, and *pgsC* that were responsible for the synthesis and transport of γ-PGA were also up-regulated by 3, 10, and 30 times, respectively (Fig. 3H). Their significant up-regulations verified the vital function of PFP in the optimized γ-PGA generation symbiont. Compared with the production of γ-PGA by microbial fermentation, the artificial symbiotic system directly converts CO_2_ and N_2_ from air to γ-PGA. The constructed solar-powered multi-organism symbiont system could synthesize high-valued products such as polypeptides, improve efficiency of synthesis, and shorten the biosynthesis cycle. Therefore, artificial symbiont as a model for biosynthesis transcends the limitations of natural synthesis.

### The multi-organism symbiont mimic system for bacitracin A synthesis

**Fig. 4.**
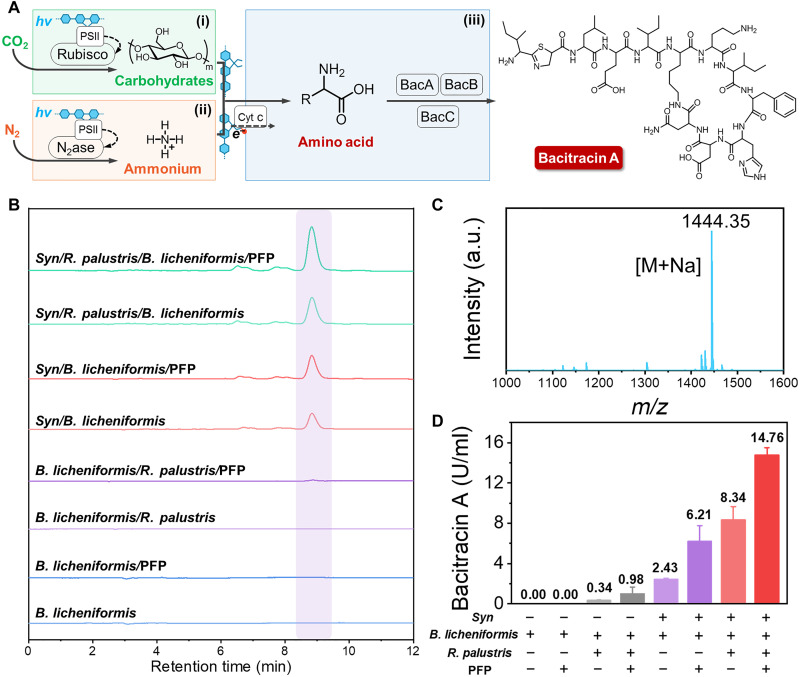
The multi-organism symbiont mimic system for synthesis of bacitracin A. (**A**) Modular assembly of bacitracin A logical biosynthetic pathway from CO_2_ and N_2_. (**B**) Analysis of different logic synthesis circuits by HPLC spectrum. (**C**) Mass spectrometry of bacitracin A produced by *Syn*/*R. palustris*/*B. licheniformis*/PFP after growing for 10 days. (**D**) Synthetic bacitracin A production of different logic synthesis circuits after growing for 10 days.

The symbiotic strategy could be extended to some more applications, such as antibacterial peptide production. In the constructed coculture system, *B. licheniformis* CICC 23642 was used as the synthesis module for bacitracin A synthesis, while *Syn* and *R. palustris* were still used to supply substrates of carbohydrates and ammonium (Fig. 4A). Meanwhile, PFP could accelerate the electronic and substance communications between species and improve the yield. As shown in Fig. 4 (B and C) and fig. S15, the mass spectrometry clearly monitored the generation of bacitracin A, and the high-performance liquid chromatography (HPLC) results could further quantify the bacitracin A concentration in the system. The final synthetic amount of bacitracin A in the symbiont of *Syn*/*R. palustris*/*B. licheniformis*/PFP was 14.76 U/ml, which was increased by 77 and 138%, respectively, compared with those of *Syn*/*R. palustris*/*B. licheniformis* and *Syn*/*B. licheniformis*/PFP (Fig. 4D). In addition, bacitracin A in the coculture system was negligible in the absence of *Syn* and PFP due to the lack of carbon source. All these results kept the similar tendency as the above symbiont of *Syn*/*R. palustris*/*B. licheniformis*/PFP for γ-PGA synthesis.

## DISCUSSION

In summary, the study constructed a solar-driven symbionts consisting of multi-organisms and conductive polymer for the selective synthesis of γ-PGA based on DISET. In the system of γ-PGA biosynthetic processes, *Syn* and *R. palustris* could fix CO_2_ and nitrogen to carbohydrate and ammonium for γ-PGA generation in *B. licheniformis*, and the by-products of acetic acid from *B. licheniformis* could be absorbed in the metabolic pathway of *R. palustris*. The assembled PFP could further optimize the photosynthetic process and photoelectron production of *Syn* and *R. palustris* to promote the generation, communication, and utilization of carbon and nitrogen source inside. PFP, with satisfying conductivity, could also contribute to a better DISET for efficient γ-PGA synthesis in the formed artificial symbiotic system. Attributing to the mutually beneficial relationships, the synthesis rate of γ-PGA was thus increased by 104% and pushed to 144.2 mg/liter with substantially up-regulated gene expression levels of *rbcL*, *prk*, *nifH*, *nifD*, *pgsB*, *pgsA*, and *pgsC*. This strategy could be further extended to synthesize peptide antibiotic bacitracin A using airborne CO_2_ and N_2_ as long as the matched functional organisms were assembled in the symbiont. Compared with the existing harsh environment of CO_2_ and N_2_ fixation, it is of more important practical significance in the field of biosynthesis to convert airborne CO_2_ and N_2_ into high-value products under normal temperature and pressure by constructing solar-powered multi-organism symbiont. In the future, element ratio, light capturing ability of materials, and directional assembly of material-microbial interface are expected to adjust microbial metabolic network in artificial symbiont to achieve specific product formation and possibly achieve higher production efficiency. The advantages of artificial symbiont integrated material/microorganism hybrid, high solar capture, efficient charge transfer, and selective biosynthesis will break through the maximum theoretical limit of light-driven biosynthesis. The proof of concept of artificial symbiont provided a revolutionary tool for the programmable biosynthesis of functional peptides and proteins prospecting broad applications on the frontier of medicine, food, energy, and environmental protection.

## MATERIALS AND METHODS

### Materials and instruments

All chemicals and solvents used in the experiments were bought from Acros, Sigma-Aldrich Chemical Company, or Beijing Chemical Works. PFP was prepared on the basis of the literature (*39*). *Synechocystis* sp. *PCC6803* was purchased from Freshwater Algae Culture Collection at the Institute of Hydrobiology. *B. licheniformis* CICC 10099 and 23642 were obtained from the China Center of Industrial Culture Collection (CICC). *R. palustris* BNCC3366448 was purchased from Beina Chuanglian Biological Technology Co. Ltd. Toray carbon paper (TGP-H-090), Ag/AgCl (saturated in KCl solution), and platinum wire electrodes were purchased from Shanghai Chuxi Industrial Co. Ltd. Amplite fluorimetric NADP^+^/NADPH ratio assay kit 15264 and enhanced ATP assay kit were purchased from AAT Bioquest and Beyotime Biotechnology, respectively.

ITC was measured on microcal VP-ITC. Electrochemical measurements were carried out on CHI1040C (Shanghai CH Instrument Co., China) and Autolab PGATAT302N (Metrohm, Switzerland). UV-visible absorption measurements were taken on a JASCO V-550 spectrophotometer. Fluorescence spectra were conducted with a Hitachi F-4500 spectrofluorometer equipped with a xenon lamp excitation source. SEM images were measured on a JSM 6700F SEM (Hitachi, Japan). AFM images were viewed with Bruker MultiMode 8. The optical source was produced by Xenon fiber optic lamp (CXE-350, Optprco, China). Chlorophyll fluorescence parameters were measured by Handy PEA chlorophyll fluorimeter and FMS-2 pulse-modulated fluorimeter (Hansatech, UK). The illumination intensity was regulated by a radiometer (Photoelectric Instrument Factory of Beijing Normal University). The quantitative measurement of glutamate was performed with an HPLC III 400 MHz HD spectrometer (Waters 2535Q system). The acetic acid was measured by gas chromatography (GC) (Agilent, GC8860) equipped with a flame ionization detector (FID). The zeta potential was measured with Malvern Zetasizer Nano ZS90 (ZEN3600). CLSM images were viewed with a CLSM (Olympus FV 1200-BX61, Japan). Ultrapure water (resistivity >18.2 megohm·cm at 25°C) obtained from a Milli-Q water purification system (Millipore Corp., Bedford, MA, USA) was used for all the experiments. All experiments and measurements were carried out at room temperature unless indicated otherwise.

### Bacterial strains and media composition

BG11 was used as the sterile medium of *Synechocystis* sp. *PCC6803* (*Syn*). The cultured medium of *R. palustris* was K_2_HPO_4_ (1.0 g/liter), MgSO_4_ (0.5 g/liter), and yeast extract (10 g/liter). The pH of medium was adjusted to 7.4 with NaOH. Under the continuous irradiation of 1500 lux white light at 25°C, the light was cycled for 12 hours and dark for 12 hours. *R. palustris* required closed tubes filled with medium for culture. The conical flasks were shaken three times a day. The cultured medium of *B. licheniformis* CICC 10099 was K_2_HPO_4_ (0.5 g/liter), MgSO_4_ (0.24 g/liter), citric acid (2.0 g/liter), ferriamine citrate (0.5 g/liter), glycerin (20 g/liter), and agar (13 g/liter), and the pH was adjusted to 7.4 with NaOH. The medium and bottles were autoclaved for 20 min at 121°C and cooled to room temperature. The bacterial powder was dissolved in sterile water in suspension form and transplanted into a solid culture tube, which was placed diagonally at 30°C for culture. After the inclined surface grew into colonies, *B. licheniformis* were scraped into a liquid culture medium without agar and cultured at 30°C, 180 rpm for 24 hours. *B. licheniformis* were washed with 1× phosphate-buffered saline (PBS) phosphoric acid buffer solution (pH 7.4) and centrifuged at 7500 rpm for 3 min. Then, optical density at 600 nm (OD_600_) was adjusted to 1.0, and the mutagenesis was carried out by UV irradiation for 30 min. The bacterial solution (100 μl) was diluted 50,000 times and taken into the prepared bacterial plate. The bacteria stick was evenly coated and grown at 30°C for 48 hours. The sticky, smooth, and opaque colonies were selected to culture in liquid medium for 24 hours and preserved at −80°C with 30% sterilized glycerin as cryoprotectant. The cultured medium of *B. licheniformis* CICC 23642 was peptone (5.0 g), beef extract (3.0 g), NaCl (5.0 g), and agar (13 g/liter), and pH was adjusted to 7.0 with NaOH. The medium and bottles were autoclaved for 20 min at 121°C and cooled to room temperature. The bacterial powder was dissolved in sterile water in suspension form and transplanted into a solid culture tube, which was placed diagonally at 30°C for culture. After the inclined surface grew into colonies, *B. licheniformis* were scraped into a liquid culture medium without agar and cultured at 30°C and 180 rpm for 24 hours. The defined photosynthesis medium (DPM) was NaNO_3_ (5 g/liter), K_2_HPO_4_ (0.04 g/liter), MgSO_4_ (0.15 g/liter), citric acid (1.0 g/liter), ferriamine citrate (0.25 g/liter), EDTANa_2_ (1 mg/liter), and Na_2_CO_3_ (0.02 g/liter). DPM was sterilized by passage through a 0.2-μm surfactant-free cellulose acetate (SFCA) filter. The concentration of *Syn* and *R. palustris* was determined by OD_730_, and the concentration of *B. licheniformis* was determined by OD_600_.

### Electrochemical measurements

Electrochemical measurements including chronoamperometry, cyclic voltammetry, and electrochemical impedance spectroscopy (EIS) were measured with a standard three-electrode system. Photocurrents and cyclic voltammetry were conducted with a CHI1040C electrochemical workstation. The process of working electrode for *Syn*/PFP or *R. palustris*/PFP electrode is as follows: 100 μl of PFP, OD_730_ = 2.0 *Syn* (100 μl) or *R. palustris*, and 40 μl of 0.5% Nafion were pipetted into the active section of carbon paper sequentially. The electrode was dried in air between settling of each liquid settled. Similarly, *Syn* or *R. palustris* electrode was prepared by adding 100 μl of deionized water, 100 μl OD_730_ = 2.0 of *Syn* or *R. palustris*, and 40 μl of 0.5% Nafion to the substrate. *Syn*, PFP, *Syn*/PFP, *R. palustris*, and *R. palustris*/PFP coated with carbon papers (1 cm^2^) were used as working electrodes. Pt and Ag/AgCl electrode were used as the counter and reference electrode, respectively. The electrolyte was PBS (pH 7.4), and the illumination intensity was 60 mW/cm^2^. The photocurrents were measured at the bias voltage of 0.2 V (versus Ag/AgCl) under periodic light (10 s) and dark (10 s). Cyclic voltammetries were measured in 0.01 M PBS by removing the oxygen (pH 7.4) solution at a scan rate of 0.1 V s^−1^. Linear sweep voltammetry was conducted with a CHI1040C electrochemical workstation at a scan rate of 50 mV s^−1^.

### EIS measurements

EIS was measured with an Autolab PGATAT302N electrochemical workstation. The process of working electrode for *Syn*/PFP or *R. palustris*/PFP electrode is as follows: 100 μl of PFP (50 μM), OD_730_ = 2.0 *Syn* (100 μl) or *R. palustris*, and 40 μl of 0.5% Nafion were pipetted into the active section of carbon paper sequentially. The *Syn*, *R. palustris*, and PFP electrode were the control group. One hundred microliters of PFP (50 μM), OD_600_ = 2.0 *B. licheniformis* (100 μl), and 40 μl of 0.5% Nafion were pipetted into the active section of carbon paper sequentially. The *B. licheniformis* electrode was the control group. The electrolyte was 1 mM Fe(CN)_6_^4−^/^3−^ solution. EIS measurements were used with a standard three-electrode system. Pt and Ag/AgCl were used as the counter and reference electrode, respectively.

### Zeta potential measurements

The *B. licheniformis*, *Syn*, and *R. palustris* samples were incubated with PFP solution (10 μM) at 10 min at 30°C. The free polymer was removed by centrifuging at 7200 rpm for 3 min. The obtained bacterial were washed with ultrapure water and then resuspended in ultrapure water for zeta potential measurements. The untreated *B. licheniformis*, *Syn*, and *R. palustris* were measured as control.

### ITC measurements

PBS, *B. licheniformis*, *Syn*, or *R. palustris* (200 μl) was added to the sample tank and stirred continuously with a blender, adding 50 μM PFP at regular intervals. During the test, the temperature was maintained at 25°C.The binding parameters were obtained by fitting the ITC curves.

### SEM measurements

*B. licheniformis* (OD_600_ = 0.2), *Syn* (OD_730_ = 0.5), *R. palustris* (OD_730_ = 0.1), and *B. licheniformis*/*Syn*/*R. palustris* were incubated with PFP solution (10 μM) at 10 min at 30°C. The unbound PFP was removed by centrifuging at 7500 rpm for 3 min. The samples were dropped on clean silicon slices and allowed to evaporate at 25°C. After the specimens were dried, 2.5% glutaraldehyde was added for fixation overnight. When drying out in the air, samples were fixed with glutaraldehyde (2.5%) in ultrapure water for 8 hours. Ethanol was added in a graded series (5, 10, 30, 50, 70, 90, and 100% for 6 min, respectively) followed by natural drying in the air. Last, the specimens were sprayed with platinum before characterization by SEM.

### CLSM characterization

*B. licheniformis* (OD_600_ = 0.2), *Syn* (OD_730_ = 0.5), *R. palustris* (OD_730_ = 0.1), and *B. licheniformis*/*Syn*/*R. palustris* were incubated with PFP solution (10 μM) at 10 min at 30°C. The unbound PFP was removed by centrifuging at 7500 rpm for 3 min. The mixtures were washed with deionized water twice and then mounted on a glass slide with a coverslip on top and examined with confocal lasers to excite PFP and *Syn*, which were 405 and 488 nm, respectively.

### Chlorophyll fluorescence kinetics measurements

The chlorophyll fluorescence parameters of *Syn* and *Syn*/PFP were measured by Handy PEA chlorophyll fluorescence meter and FMS-2 pulse modulation fluorescence meter. *Syn* and *Syn*/PFP (1.5 ml) were added to the sample bottle, and the photosynthetic parameters were measured. Then, after the dark adaption for 30 min, the PSII reaction center was fully opened, and FMS-2 and Handy PEA fluorometer were measured.

### AFM measurements

The *B. licheniformis* (OD_600_ = 0.2), *Syn* (OD_730_ = 0.5), and *R. palustris* (OD_730_ = 0.1) samples were mixed evenly and incubated with PFP solution (10 μM) at 10 min at 30°C. The unbound PFP was removed by centrifuging at 7500 rpm for 3 min. Then, 10 μl of suspensions was spread on a mica sheet. The samples were imaged with AFM, and the untreated mixed bacteria were imaged in the same conditions. In addition, to test the surface potential of the samples, it is necessary to coat the mica with conductive platinum before sample preparation.

### The growth curve of microbial

*B. licheniformis* (OD_600_ = 0.1), *Syn* (OD_730_ = 0.1), and *R. palustris* (OD_730_ = 0.3) were diluted for the growth curve in graded PFP concentration series (0, 5, 10, and 20 μM). *Syn* and *R. palustris* were cultured in an illumination incubator, with continuous irradiance of white light (1500 lux) on a cycle of 12-hour light and 12-hour dark. *B. licheniformis* was cultured at 30°C. The concentration of samples was determined with an Evolution 201 spectrophotometer.

### Quantification of γ-PGA

After the fermentation, the fermented liquid was centrifuged at 8000 rpm for 5 min to collect the supernatant, and anhydrous ethanol with three times the volume was added. The γ-PGA precipitate was obtained by lyophilization overnight and by centrifugation at 11,000 rpm for 10 min. The precipitation was dissolved into ultrapure water and centrifuged at 11,000 rpm for 10 min to obtain the supernatant as γ-PGA solution. The turbidity of γ-PGA reaction with cetyltrimethylammonium bromide (25 g/liter)–2% NaOH solution could be reflected by the absorbance of the reaction system, and then the content of γ-PGA can be calculated by the linear relationship between turbidity and γ-PGA concentration.

### Construction of coculture system

*B. licheniformis* CICC 10099 or 23642 (OD_600_ = 0.2), *Syn* (OD_730_ = 0.5), *R. palustris* (OD_730_ = 0.1), *Syn*/*B. licheniformis*, *B. licheniformis*/*R. palustris*, *Syn*/*R. palustris*, and *Syn*/*R. palustris*/*B. licheniformis* were incubated with PFP solution (10 μM) for 10 min at 30°C. Untreated samples of various bacteria were used as controls. The coculture systems were cultured in an illumination incubator with DPM, with a continuous irradiance of white light (1500 lux) on a cycle of 12-hour light and 12-hour dark for 10 days at 25°C.

### Quantification of by-products

Samples were taken from the six-hole plate and filtered through a 0.22-μm cellulose membrane filter. The CH_3_COOH solution was measured by GC and ^1^H-NMR in D_2_O. CH_3_COOH standard curve (1, 2, 5, 10, 50, and 100 mM) was detected through GC. The amount of CH_3_COOH was determined using a GC equipped with a FID and a DB-WAX column. N_2_ was used as mobile phase. The initial temperature of the capillary column was 150°C, and the retention time was 2 min. In addition, the injection temperature and FID temperature were 200°C.

### Measurements of interspecific electron transport

The process of working electrode for *Syn*/PFP or *R. palustris*/PFP electrode is as follows: 100 μl of PFP (50 μM), OD_730_ = 2.0 *Syn* (100 μl) or *R. palustris*, and 40 μl of 0.5% Nafion were pipetted into the active section of carbon paper sequentially. The electrode was dried in air between each liquid settled. Similarly, the *Syn* or *R. palustris* electrode was prepared by adding 100 μl of deionized water, OD_730_ = 2.0 *Syn* (100 μl) or *R. palustris*, and 40 μl of 0.5% Nafion to the substrate. *Syn*, *Syn*/PFP, *R. palustris*, and *R. palustris*/PFP coated on carbon papers (1 cm^2^) were used as working electrodes. The carbon paper was incubated in *B. licheniformis* and *B. licheniformis*/PFP for 24 hours at 30°C to form the biofilm. The control group of dead bacteria was obtained by ultrasonic crushing, and other operations were the same as the above method. Pt and Ag/AgCl electrode were used as the counter and reference electrode, respectively. The electrolyte was PBS (pH 7.4), and the illumination intensity was 60 mW/cm^2^. The polarization curves of the cells by two kinds of bacteria were measured. The power curves were obtained by calculating the polarization curve.

### Measurement of carbohydrate and NH_4_^+^

The cocultured samples were centrifuged at 8000 rpm for 10 min to obtain the supernatant. The supernatant (0.5 ml) was added into the 2.5-ml anthrone solution (1 mg/ml/80% H_2_SO_4_). The mixture was boiled in a boiling bath for 10 min, and the absorbance at 625 nm was detected. The carbohydrate content was calculated via the standard curve (a series concentration of glucose solutions). NH_4_^+^ was measured by a Solarbio ammonium nitrogen kit.

### Measurement of intracellular ATP and NADPH/NADP^+^ ratio

The physically separated system was used by dialysis membrane. The physically separated setup was a semipermeable membrane between bacterial species. Five milliliters of *Syn* (OD_730_ = 1.0) or *R. palustris* (OD_730_ = 1.0) was incubated with PFP (10 μM) at room temperature for 10 min. As control, 5 ml of cell resuspensions was added to equal volume of ultrapure water. *B. licheniformis* (OD_600_ = 0.2), *Syn* (OD_730_ = 0.5), and *R. palustris* (OD_730_ = 0.1) were mixed evenly and incubated with PFP solution (10 μM) for 10 min at 30°C. Untreated samples of various bacteria were used as controls. The prepared samples were measured under the light intensity of 10 mW cm^−2^ for 60 min. The samples (5 ml) were collected by centrifuging at 7500 rpm for 3 min. A total of 0.5 ml of cell lysis buffer was added, and ultrasound was performed for 30 min. The NADPH/NADP^+^ ratio and ATP were measured by an Amplite fluorimetric NADPH/NADP^+^ ratio assay kit and an enhanced ATP bioluminescent assay kit (Beyotime Biotechnology), respectively.

### Photosynthetic efficiency calculations

The quantum yield was determined by comparison of the initial rate of products with the measured photon flux.

The photosynthetic efficiencies (PE) equation is as follows

$$\begin{array}{c} 6n\;{\mathrm{CO}}_{2}+24n\;H^{+}+24n\;e^{-}+24n\;hv\to {\left(C_{6}H_{12}O_{6}\right)}_{n}+6n\;H_{2}O\\ N_{2}+8H^{+}+8e^{-}+8\;hv\to 2\;{NH}_{3}+H_{2}\\ \mathrm{PE}\;\%=\frac{m\times C\times N_{A}\times V}{\phi_{\mathrm{ph}}\times t\times A}\times 100\% \end{array}$$

where *m* is the number of electrons for per mole product, *C* is the carbohydrate or NH_4_^+^ concentration, *V* is the total suspension volume, ϕ_ph_ cm^−2^ s^−1^ is the photo flux, *A* is the area of illumination, *t* is the reaction time, and *N*_A_ is Avogadro’s number.

The following calculation example is based on the data

*V* = 5 ml, ϕ_ph_ = 1.144 × 10^15^ s^−1^ cm^−2^, *A* = 10 cm^2^, *N*_A_ = 6.022 × 10^23^, *t* = 120 hours × 3600 s = 432,000 s, and *m* = 24 (carbohydrate) or 8 (NH_4_^+^).

### Quantitative reverse transcription polymerase chain reaction

SYBR Green I real time PCR was used to detect the mRNA transcription of target genes in the mixed samples of bacteria and algae. The specific primers of eight genes (two critical enzymes RuBisCO and PRK in *Syn*, three nitrogen-fixing key genes of *R. palustris*, and three γ-PGA synthesis key genes of *B. licheniformis*) were designed (table S3). The housekeeping gene β*-actin* and *16*S* RNA* served as internal control.

### Detection of bacitracin A

After the fermentation, the fermented liquid was centrifuged at 8000 rpm for 5 min to collect the supernatant, and anhydrous ethanol with nine times the volume was added. After centrifugation at 11,000 rpm for 10 min, the supernatant was freeze-dried and resuspended in 1 ml of ultrapure water. Then, HPLC quantified the production of bacitracin A. The metabolites were analyzed by HPLC with a UV detector equipped with an XBridge BEH C18 analytical column (5 μm, 4.6 mm × 250 mm). The system was operated in isocratic mode using 35% water/60% methanol/5% acetonitrile as mobile phase at a flow rate of 1 ml min^−1^. Standard curves for each metabolite were constructed with pure standards. For bacitracin A, the retention time was observed at around 9.0 min with a detection wavelength at 210 nm.
