# Supplementary material for: Solar-powered multi-organism symbiont mimic system for beyond natural synthesis of polypeptides from CO2 and N2
Source: Sci Adv. 2023 Mar 15;9(11):eadf6772. doi: 10.1126/sciadv.adf6772 (PMC10017035; doi:10.1126/sciadv.adf6772)
Supplement: Supplementary file 1 — Figs. S1 to S15 Tables S1 to S3 [file sciadv.adf6772_sm.pdf]

Science Advances
AAAS

Supplementary Materials for

Solar-powered multi-organism symbiont mimic system for beyond natural synthesis of polypeptides from CO_2 and N_2

Wen Yu *et al.*

Corresponding author: Shu Wang, wangshu@iccas.ac.cn; Haotian Bai, baihaotian@iccas.ac.cn

Sci. Adv. **9**, eadf6772 (2023)
DOI: 10.1126/sciadv.adf6772

This PDF file includes:

Figs. S1 to S15
Tables S1 to S3

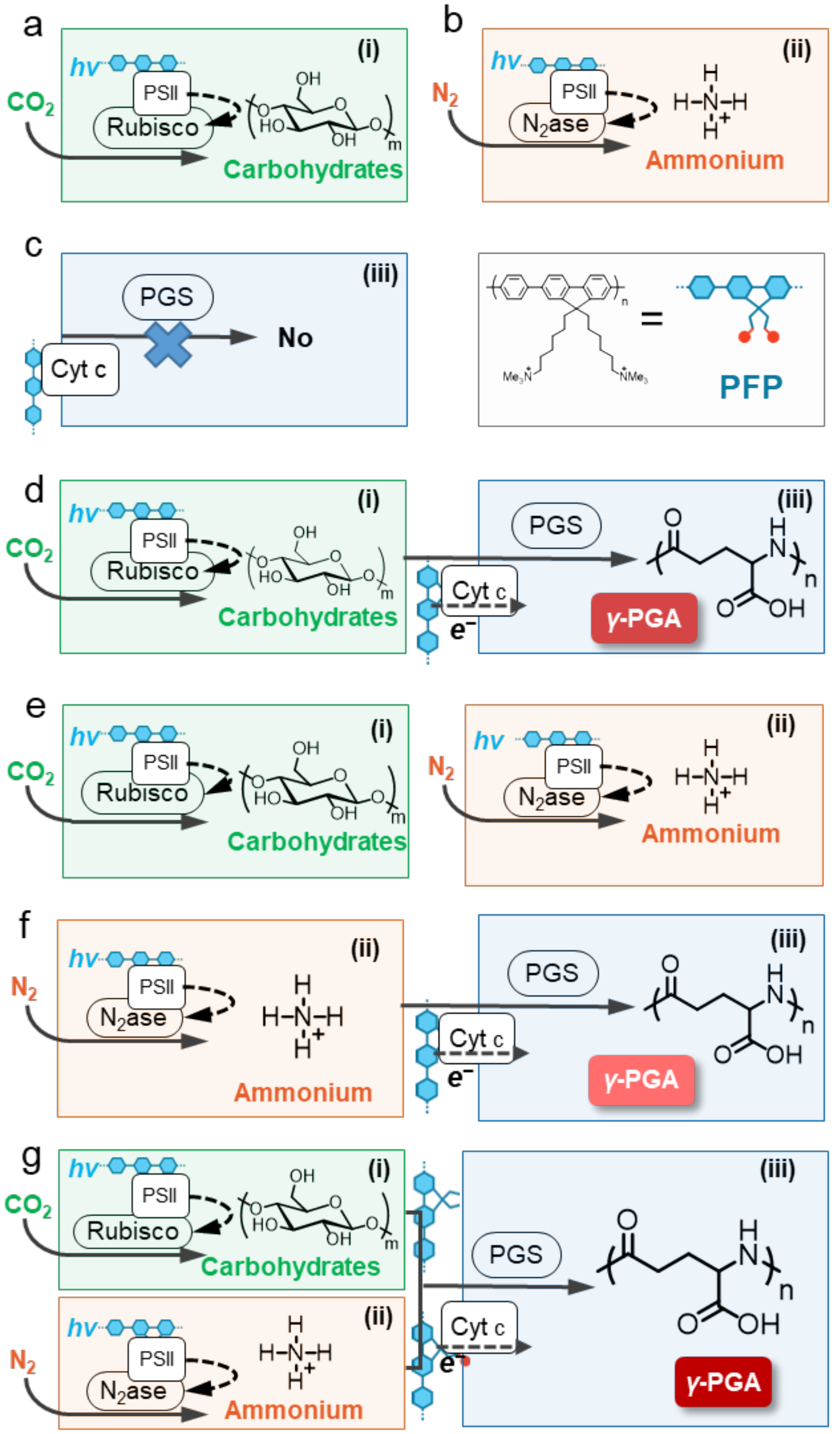

Fig. S1. The logical biosynthetic circuit from CO_2 and N_2. (a-g) Design and modular assembly of logical (digital) biosynthesis pathway from CO_2 and N_2. (i) Reduction of CO_2 to carbohydrates by *Syn*. (ii) Fixtion of N_2 to ammonium by *R. palustris*. (iii) γ-PGA synthesis from carbohydrates and ammonium by *B. licheniformis*.

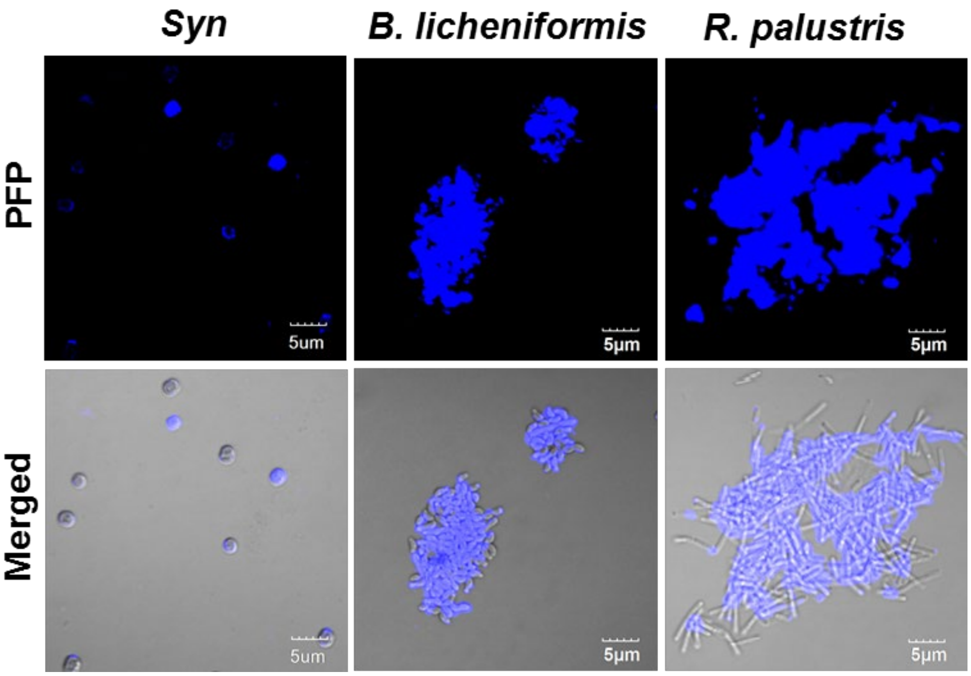

Fig. S2. CLSM images of *Syn*, *B. licheniformis* and *R. palustris* incubated with PFP. The blue fluorescence is from PFP (λ_{ex} = 405 nm, λ_{em} = 425-475 nm).

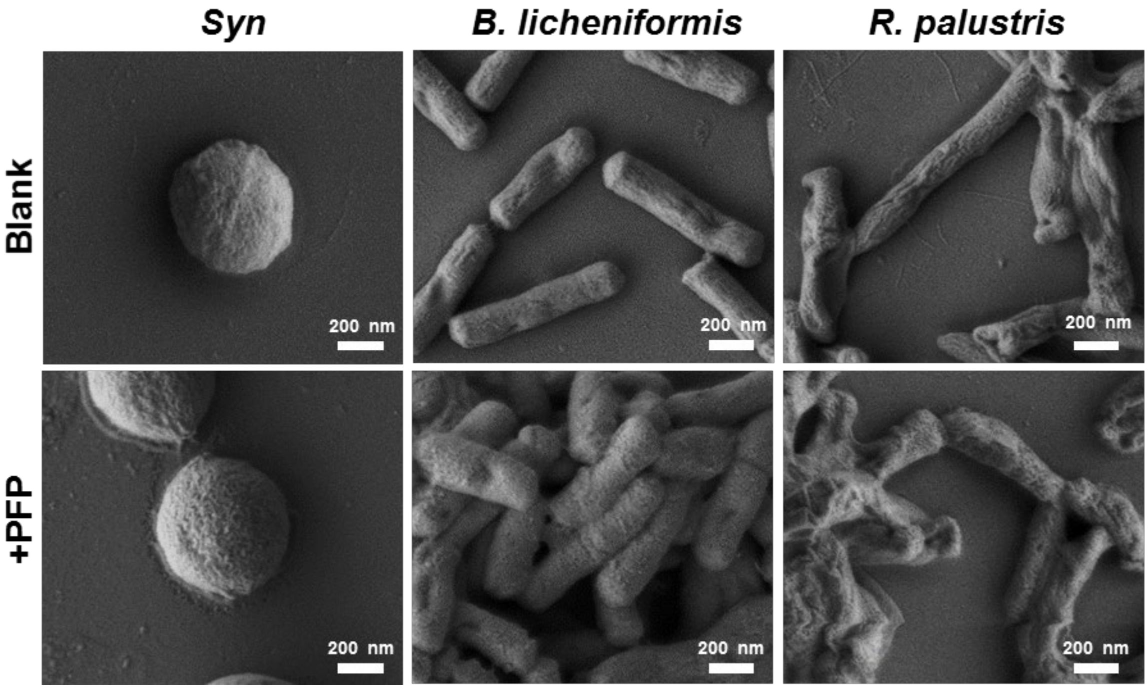

Fig. S3. SEM images of *Syn*, *Syn*/PFP, *B. licheniformis*, *B. licheniformis*/PFP, *R. palustris* and *R. palustris*/PFP, respectively.

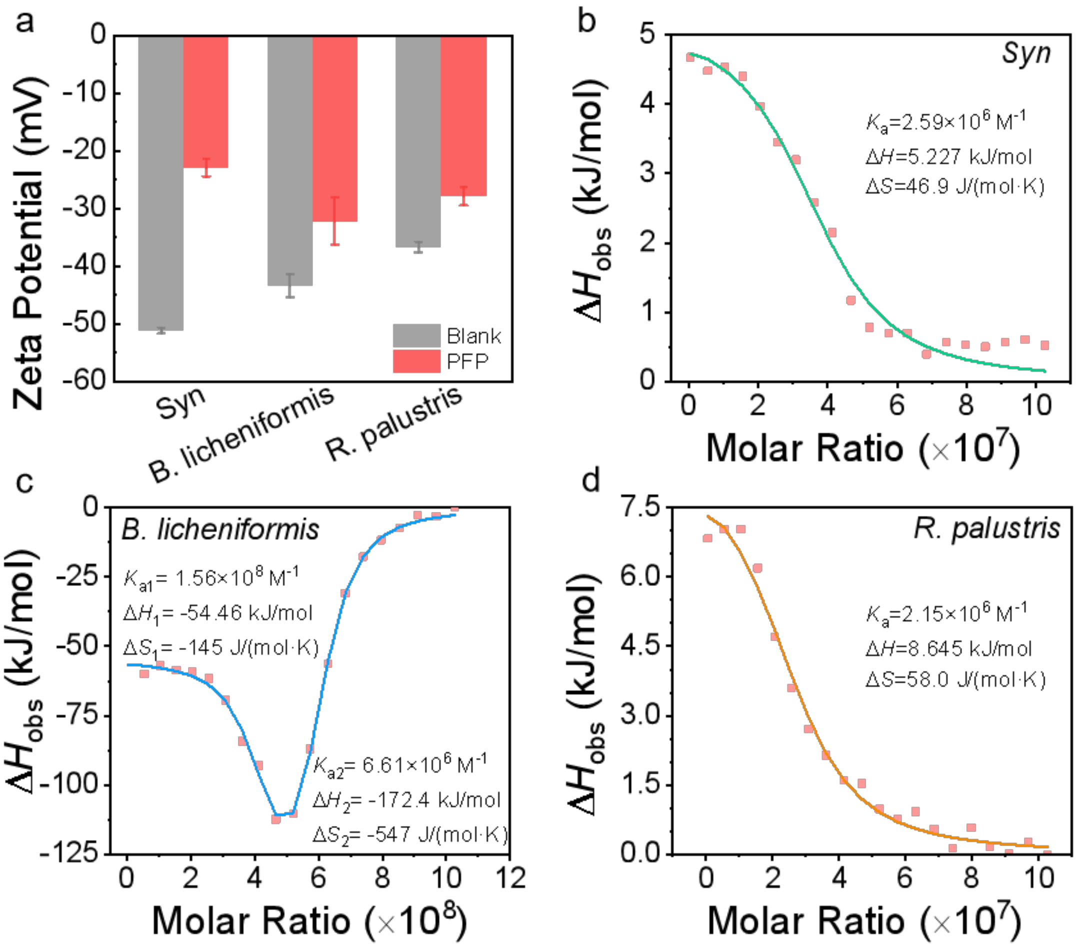

Fig. S4. Assembly of PFP and multi-organisms. (a) Zeta potentials of *Syn*, *Syn*/PFP, *B. licheniformis*, *B. licheniformis*/PFP, *R. palustris* and *R. palustris*. (b), (c) and (d) ITC curves of the titration of PFP (50 μM) into *Syn*, *B. licheniformis* and *R. palustris*, respectively.

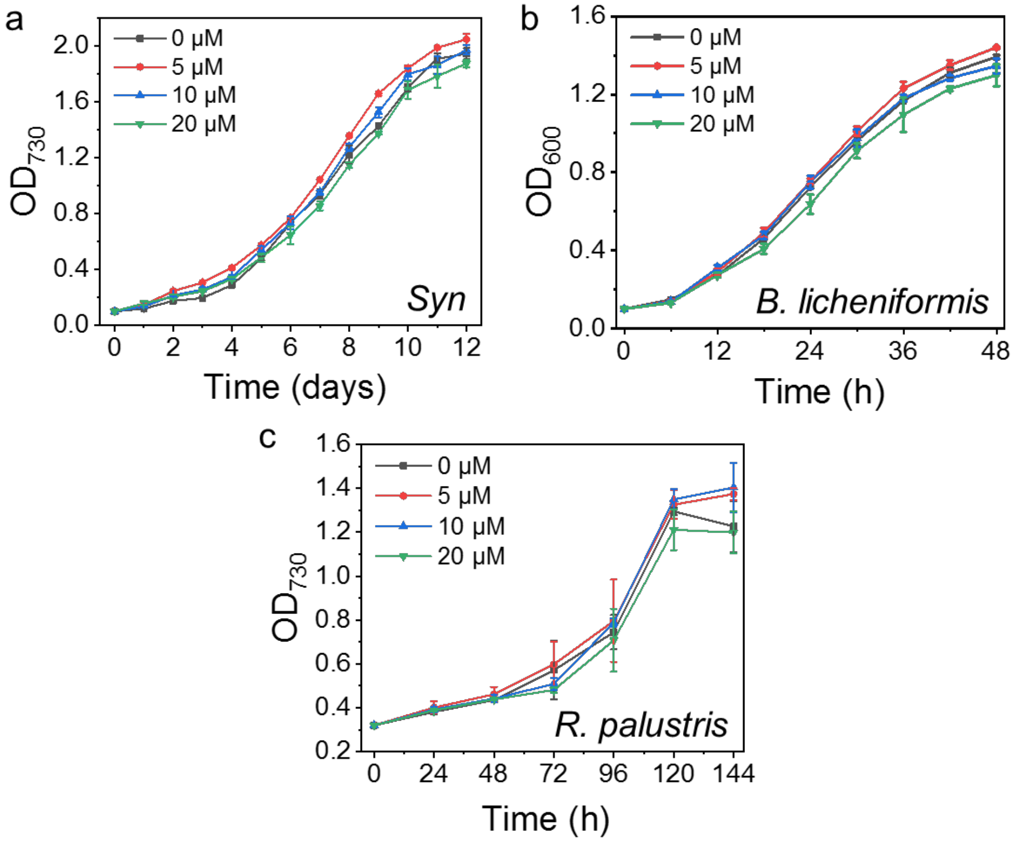

Fig. S5. The biocompatibility of PFP in microbial systems. (a) The optical density variation at 730 nm of *Syn* and *Syn*/PFP during growth process in the illumination incubator every 2 days. (b) The optical density variation at 600 nm of *B. licheniformis* and *B. licheniformis*/PFP during growth process in the illumination incubator every 6 h. (c) The optical density variation at 730 nm of *R. palustris* and *R. palustris*/PFP during growth process in the illumination incubator every 24 h. Error bars correspond to the standard deviation.

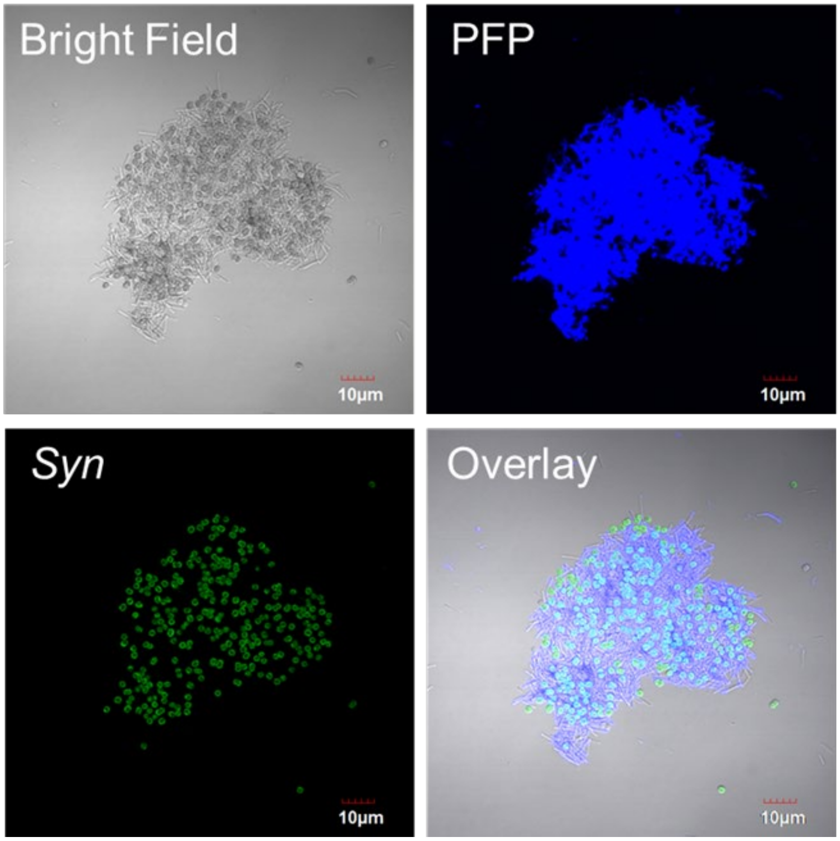

Fig. S6. CLSM images of *Syn/R. palustris/B. licheniformis* incubated with PFP. The green fluorescence is from *Syn* (λ_{ex} = 488 nm, λ_{em} = 650-750 nm), blue fluorescence is from PFP (λ_{ex} = 405 nm, λ_{em} = 425-475 nm).

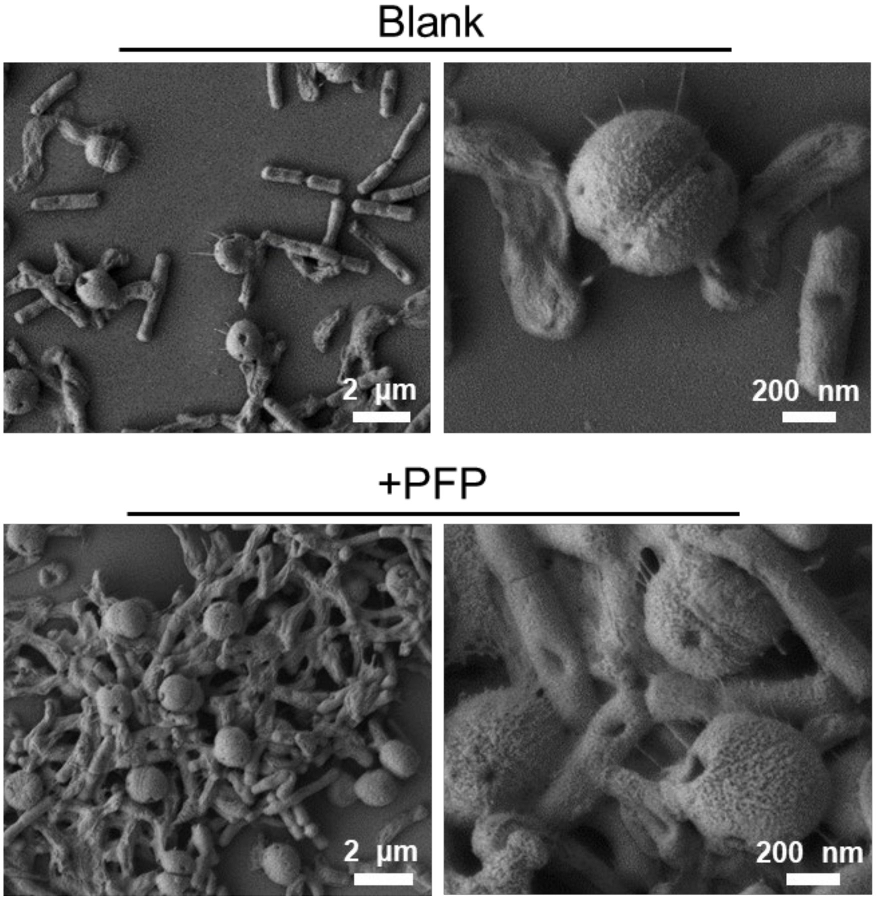

Fig. S7. SEM images of *Syn/R. palustris/B. licheniformis* and *Syn/R. palustris/B. licheniformis*/PFP, and the enlarged images of them, respectively.

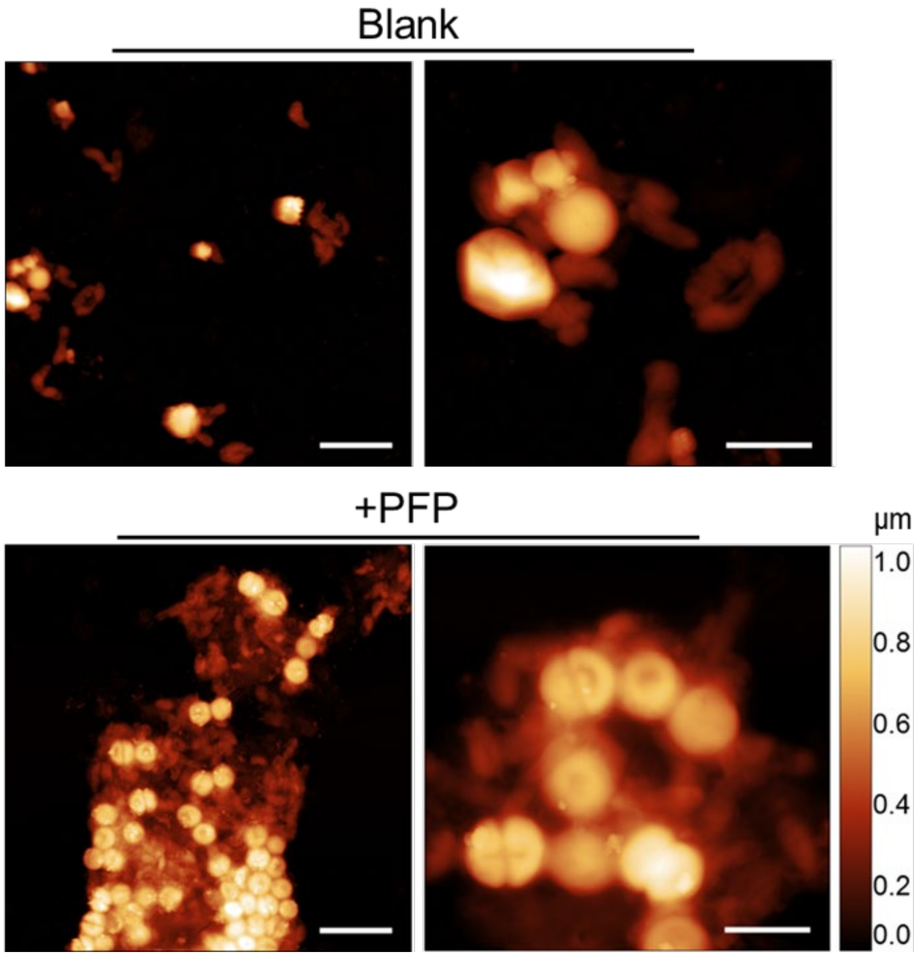

Fig. S8. AFM images of *Syn/R. palustris/B. licheniformis* and *Syn/R. palustris/B. licheniformis*/PFP (Scale bar = 5 μm), and the enlarged images of them (Scale bar = 2 μm), respectively.

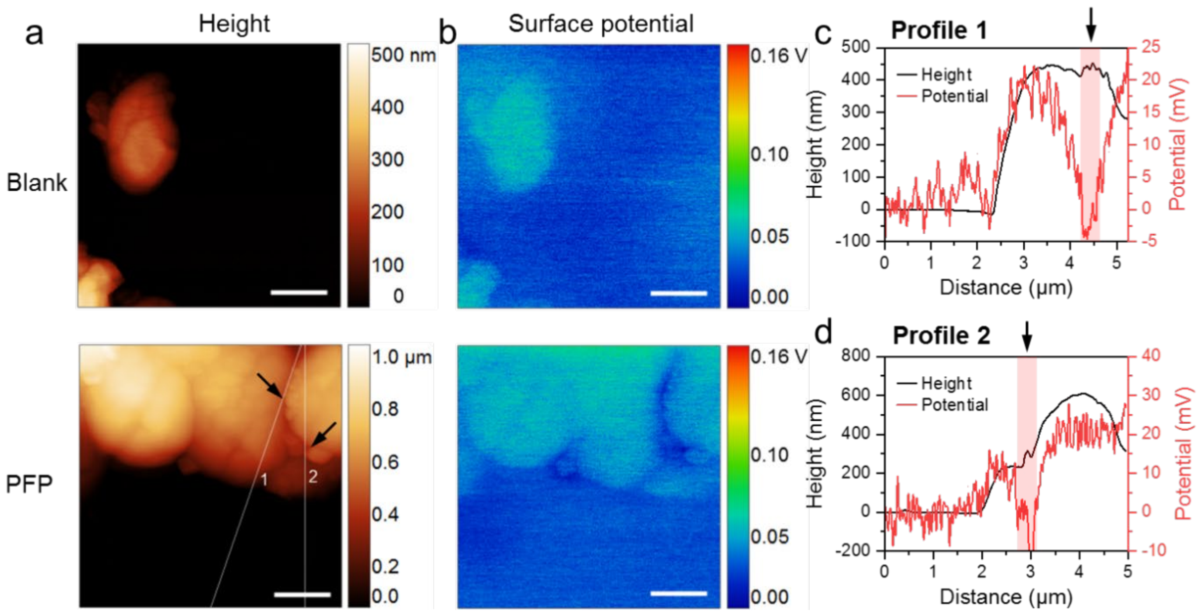

Fig. S9. The height and surface potential characterization of multi-organism symbiont mimic system. (a) Height images of *Syn/R. palustris/B. licheniformis* and *Syn/R. palustris/B. licheniformis*/PFP. (b) Surface potential images of *Syn/R. palustris/B. licheniformis* and *Syn/R. palustris/B. licheniformis*/PFP according to (a). (Scale bar = 1 μm) (c) and (d) Representative height and surface potential profile along the white line in (a), arrow indicate the locations of PFP.

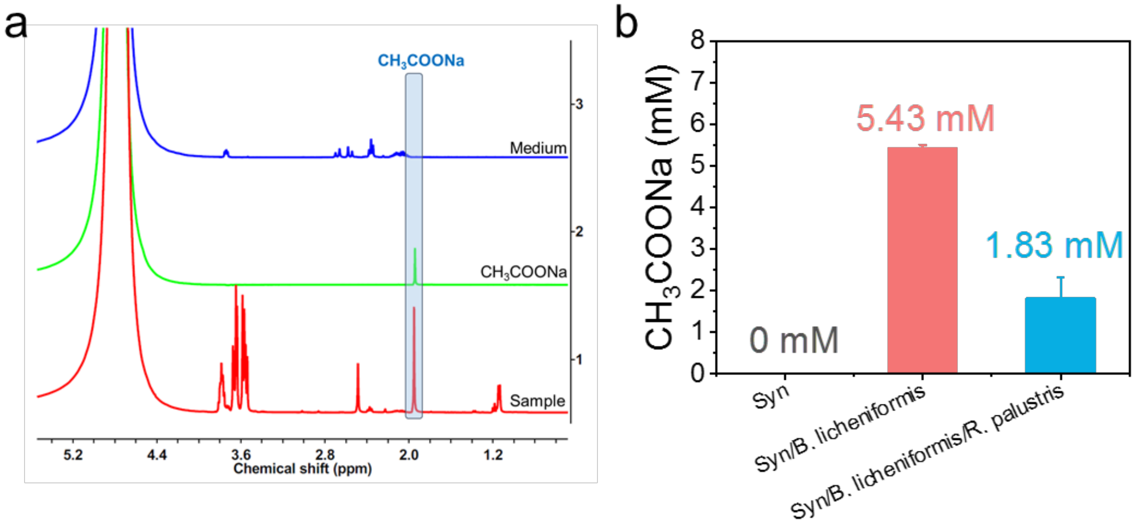

Fig. S10. Qualitative and quantitative characterization of by-products in the system. (a) ^{1}H-NMR Spectrum of co-cultured Products. (b) The CH_3COONa concentration of byproduct of co-culture system.

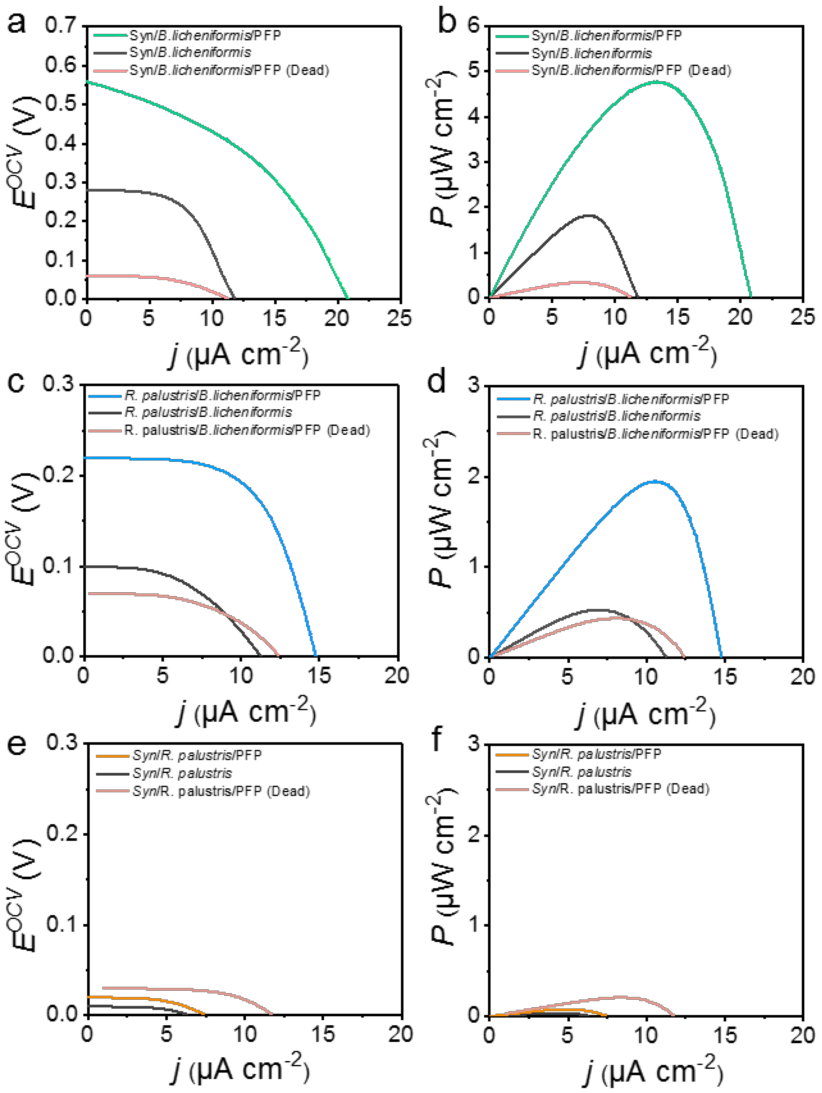

Fig. S11. Augmentation of PFP on electron transport among multi-organism. (a) and (b) polarization and power-density curves with *Syn* or *Syn*/PFP electrode as the anode, *B. licheniformis* or *B. licheniformis*/PFP electrode as the cathode. (c) and (d) polarization and power-density curves with *R. palustris* or *R. palustris*/PFP electrode as the anode, *B. licheniformis* or *B. licheniformis*/PFP electrode as the cathode. (e) and (f) polarization and power-density curves with *Syn* or *Syn*/PFP electrode as the anode, *R. palustris* or *R. palustris*/PFP electrode as the cathode.

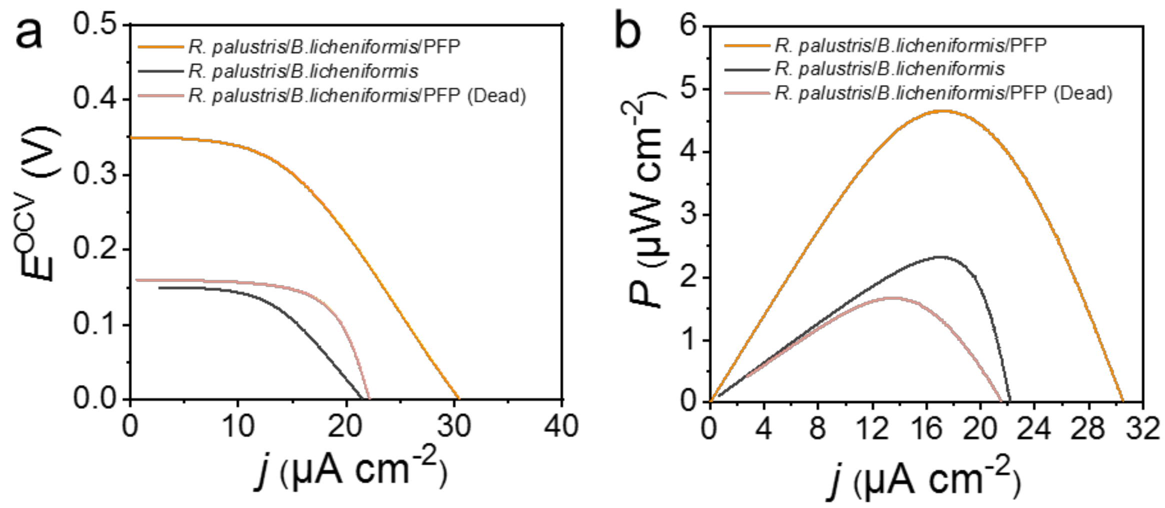

Fig. S12. Augmentation of PFP on electron transport among multi-organism in the presence of CH_3COOH. (a) and (b) polarization and power-density curves with *R. palustris* or *R. palustris*/PFP electrode as the anode, *B. licheniformis* or *B. licheniformis*/PFP electrode as the cathode in 10 mM CH_3COOH.

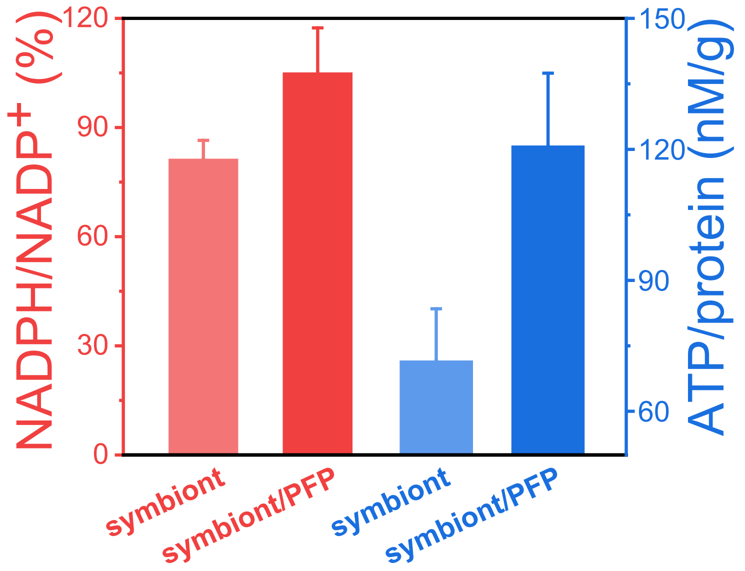

Fig. S13. The NADPH/$NADP^+$ ratio and ATP contents of co-culture systems, respectively.

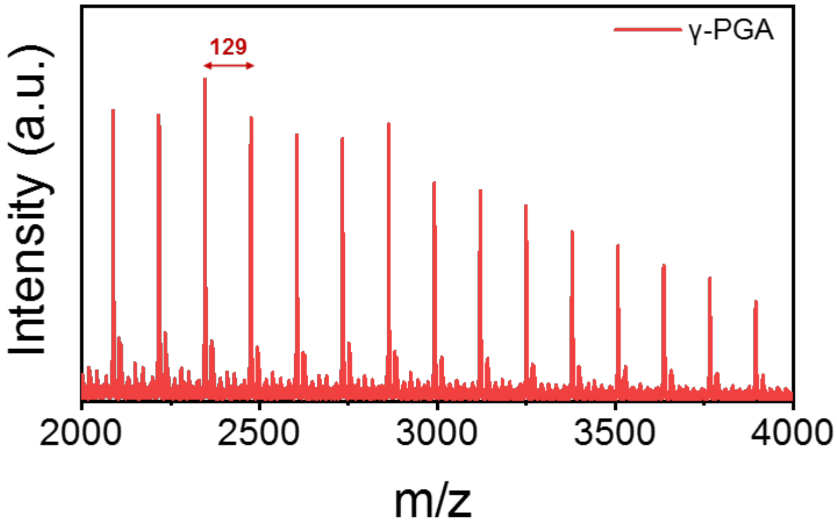

Fig. S14. The mass spectrometry of γ-PGA standard.

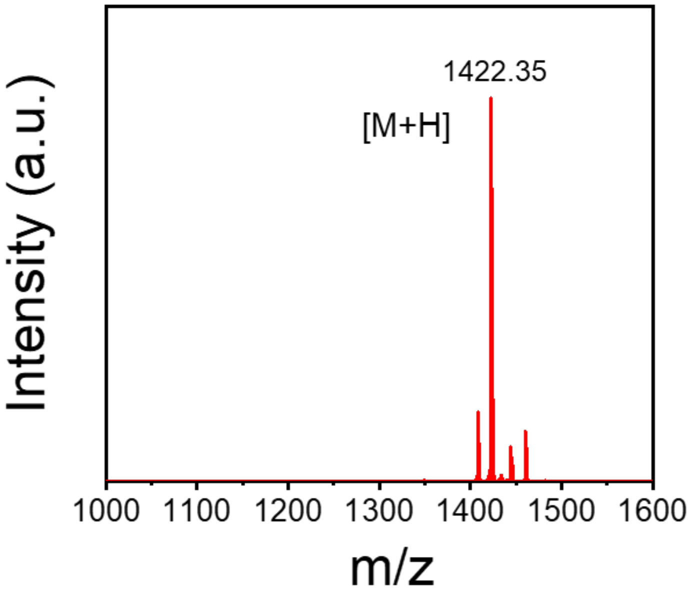

Fig. S15. The mass spectrometry of bacitracin A standard.

Table S1. Synthetic γ-PGA production of co-cultured system with different components after growing 10 days.

	Blank (mg/L）	+PFP (mg/L）
Syn	**0**	**0**
B. licheniformis	**10.72**	**13.66**
R. palustris	**0**	**0**
Syn/B. licheniformis	**55.92**	**97.78**
Syn/R. palustris	**0**	**0**
R. palustris/B. licheniformis	**19.68**	**23.07**
Syn/R. palustris/B. licheniformis	**70.66**	**144.2**

Table S2 Synthetic products of photosynthetic efficiencies in different processes.

	Without PFP	With PFP
Photosynthetic efficiency of N_2 conversion to NH_4^+	0.20%	0.31%
Photosynthetic efficiency of CO_2 conversion to carbohydrate	1.97%	2.23%
Conversion rate of γ-PGA	36%	64%
Photosynthetic efficiency of CO_2 conversion to γ-PGA	0.71%	1.43%

Table S3 Primers for quantitative reverse transcription polymerase chain reaction detection of expression genes.

Gene	Primer Sequence (5'to3')
prk	AGGAGAAGGCGGTGACTG
	GACGTGGTTGTAGATGGG
rbcL	AAACTGGTGAAATTAAAGGGC
	CACGGTGAATGTGTAAAAGAA
β-Actin	TTTGACTCAACACGGGAAAAC
	CAACCCACCAACTAAGAACGG
nifH(nifHa)	AAAGGYGGWATCGGYAARTCCACCAC
	TTGTTSGCSGCRTACATSGCCATCAT
nifD	ACCCAGTCACGAACCATGTC
	CAGTTGTACCTGTGCGTTGC
nifK	AACCGACAAAGCTAGGGGTG
	GCTGTCTGCACCACCTGTAT
pgsB	CGGAGAGCAGAAGGAGGTTA
	CCAAAGTCGGTCCCATCAC
pgsA	AAGTCGTCGCAGCCAACA
	CCGAACGGGAAGGATACA
pgsC	TATCGCCCTCATTTTAGGAGT
	AAAAGAACGAGCAGCATGAA
16sRNA	CCTACGGGAGGCAGCAG
	ATTACCGCGGCTGCTGGCA
